# 26S Proteasome Activity Is Down-Regulated in Lung Cancer Stem-Like Cells Propagated *In Vitro*

**DOI:** 10.1371/journal.pone.0013298

**Published:** 2010-10-11

**Authors:** Jing Pan, Qi Zhang, Yian Wang, Ming You

**Affiliations:** Department of Surgery, Siteman Cancer Center, Washington University School of Medicine, St. Louis, Missouri, United States of America; National Cancer Institute, NIH, United States of America

## Abstract

Cancer stem cells (CSCs) are a small subset of cancer cells capable of self-renewal and tumor maintenance. Eradicating cancer stem cells, the root of tumor origin and recurrence, has emerged as one promising approach to improve lung cancer survival. Cancer stem cells are reported to reside in the side population (SP) of cultured lung cancer cells. We report here the coexistence of a distinct population of non-SP (NSP) cells that have equivalent self-renewal capacity compared to SP cells in a lung tumor sphere assay. Compared with the corresponding cells in monolayer cultures, lung tumor spheres, formed from human non-small cell lung carcinoma cell lines A549 or H1299, showed marked morphologic differences and increased expression of the stem cell markers CD133 and OCT3/4. Lung tumor spheres also exhibited increased tumorigenic potential as only 10,000 lung tumor sphere cells were required to produce xenografts tumors in nude mice, whereas the same number of monolayer cells failed to induce tumors. We also demonstrate that lung tumor spheres showed decreased 26S proteasome activity compared to monolayer. By using the ZsGreen–cODC (C-terminal sequence that directs degradation of Ornithine Decarboxylase) reporter assay in NSCLC cell lines, only less than 1% monolayer cultures were ZsGreen positive indicating low 26S proteasome, whereas lung tumor sphere showed increased numbers of ZsGreen-positive cells, suggesting the enrichment of CSCs in sphere cultures.

## Introduction

Cancer stem cells (CSCs) are a small reservoir of self-sustaining cells with the exclusive ability for self-renewal and tumor maintenance [1]. Only a small subset of cancer cells within a tumor have the characteristics of stem cells, and can thus initiate a tumor when transplanted [3], [4]. Putative CSCs in acute myelogenous leukemia (AML) were first isolated in 1994 [5]. With the advances in fluorescence activated cell sorting (FACS), and newly identified cell surface markers such as Sca-1, and CD133, CSCs can be isolated not only from blood cancers, but also from solid tumors [1].

CSCs in human breast carcinoma have been identified as a rare population of CD44^+^/CD24^−/low^/ESA^+^ cells [6]. 100 of these cells were able to form new tumors, whereas other tumor cells failed to form tumors in SCID mice. Breast CSCs were also indentified as CD44^+^/CD24^−^/Oct-4^+^ by Ponti *et al*. in 2005 [7]. CSCs in colon cancer were found to be a less than 2.5% of the population with positive CD133 expression [8], and recently defined as CD44^+^/CD166^+^/ESA^+^ [9]. Bronchioalveolar stem cells (BASCs) were recently identified as the putative stem cell population for lung adenocarcinoma, and could be isolated as CD45^−^/Pecam^−^/Sca-1^+^/CD34^+^ cells by FACS [10]. CSCs in small cell and non-small cell lung cancer were also reported as a rare population of undifferentiated CD133^+^ cells [11]. These were able to self-renew as tumor spheres in serum-free medium, and generate tumor xenografts phenotypically indistinguishable from the original tumor, when injected in SCID mice.

An alternative approach for isolating stem cell populations is through enrichment of side population (SP) cells [2]. SP cells are identified by the capacity to efflux Hoechst dye, a process mediated by the ATP-binding cassette transporter breast cancer resistance protein (Bcrp-1) [12]. Since Goodell *et al*. first reported that the SP is enriched in hematopoietic stem cells (HSCs)[13], SP cells have been identified in a variety of normal and malignant tissues including the lung [14]. Bcrp-1 deficient mice lacked the SP in bone marrow yet had no substantial hematopoietic abnormalities [15]. Thus, the precise functional relationship between the SP phenotype and stem cell function remains unclear. Both SP and NSP fractions from the DAOY medulloblastoma cell line were capable of reconstituting the original parental population, and only slight clonogenic enrichment was observed in the SP fraction [16].

Hematopoietic stem cells (HSCs) are reported to reside in the SP fraction of adult mouse bone marrow (BM), however, a recent report showed the coexistence of NSP HSCs that do not significantly differ from SP HSCs in numbers, and self-renewal capacity [17]. In addition, the CD34^−/low^c-Kit^+^Sca-1^+^ lineage marker^−^ cell population, which is highly enriched in mouse HSCs, was almost equally divided into the SP and NSP cells. These were in a quiescent state and showed uniform cell-cycle kinetics, regardless of whether they were in the SP or NSP [17]. Further characterization of the SP in cancer cells is therefore required to fully assess the role these cells play in lung cancer.

Recent findings showed that chemotherapeutic drugs, such as doxorubicin and cisplatin, could induce the propagation of CSCs *in vitro* [18]. These cells maintained their self-renewal capacity as demonstrated by growth of tumor spheres *in vitro*, and had high tumorigenic and metastatic potential following inoculation into SCID mice. Chemotherapeutic drugs, such as cisplatin and ifosfamide, are also known to depress the expression of proteasomal subunits [19], and down-regulate ubiquitin-proteasome-dependent proteolysis [20]. Reduced 26S proteasome activity was found to be a unique feature of CICs (cancer initiating cells) in glioma and breast cancer, that could be used to track CSCs *in vivo* [21]. However, Benzyloxicarbonyl-Leu-Leu-Nle-CHO (LLNle), a γ-secretase inhibitor, was reported to effectively kill human glioblastoma tumor–initiating cells (GBM TIC) *in vitro* by inhibition of the 26S proteasome. Therefore, further elucidation of the 26S proteasome activity is required in order to fully determine its role in CSCs and anticancer therapy. Ultimately, this may aid in the identification of novel targets for future therapeutic intervention.

In the present study, we investigated whether lung CSCs solely reside in the side population and whether lung tumor spheres with self-renewal capacity from NSCLC cell lines could be a source for enrichment of lung CSCs.

## Methods

### Cell Culture

Human NSCLC cell lines A549 and H1299 were purchased from American Type Culture Collection (Manassas, VA), and cultured in RPMI 1640 media with 10% fetal bovine serum (FBS) (Gibco) and penicillin and streptomycin cocktail (Gibco). 293 FT cells were purchased from Invitrogen (Carlsbad, CA), and cultured in Dulbecco's Modified Eagle Medium (DMEM) (Gibco) with 10% FBS and penicillin and streptomycin cocktail. All cells were cultured in a humidified incubator at 37°C at 5% CO_2_.

### Staining of Cells with Hoechst 33342

Methods for identification of SPs were adapted from previous reports [22], [23]. Briefly, cells were trpsinized and resuspended at 1×10^6^ cells/ml in DMEM with high glucose, 2% (v/v) FBS, and 10 mM N-(2-hydroxyethyl) piperazine-N'-(2-ethanesulfonic acid) (HEPES)), incubated with 5 µg/ml Hoechst 33342 dye (with or without Fumitremorgin C (MP Biomedicals, Eschwege, Germany) for 90 min in a 37°C water bath, and tubes were gently inverted every 20 min to discourage cell settling and clumping. Cells were centrifuged at 375×g for 6 min at 4°C, and resuspended in an appropriate volume of cold Hank's balanced salt solution (HBSS) with 2% (v/v) FBS. The cells remained at 4°C for the remainder of the experiment. For dead cell discrimination, 2 µg/mL of propidium iodide (PI) were added immediately prior to flow cytometry. Cell samples were analyzed on a MoFlo (Beckman Coulter) cell sorter, and emission was collected through a 610 nm long pass dichroic mirror (DCLP) to a 620 nm long pass (LP) filter for the Hoechst red collection and a 424/44 nm band pass (BP) filter for the Hoechst blue collection. The side population “SP” was identified as a group of cells able to exclude the Hoechst dye, a characteristic abolished with 10 µM Fumitremorgin C treatment [22].

### Sphere Formation Assay

Lung tumor spheres were isolated through culture at low density in serum-free culture condition as previously described, which allowed the selection of undifferentiated cancer stem and progenitor cells, while serum-dependent differentiated tumor cells and non-transformed accessory cells were negatively selected [8], [11], [24]. A549 and H1299 lung cancer cells were plated at clonal density in serum free DMEM–F12 medium supplemented with, 5 mM HEPES, 0.1% sodium bicarbonate, 0.4% BSA, glutamine and antibiotics (Gibco-Invitrogen), and containing 20 ng/ml EGF and 10 ng/ml bFGF. Untreated tissue culture flasks were used to reduce cell adherence and support growth as undifferentiated tumor spheres. Medium was replaced or supplemented with fresh growth factors twice a week until cells started to grow and form floating aggregates. Tumor spheres were collected with a 100 µm cell strainer (BD, Bedford, MA), and expanded by enzymatic and mechanical dissociation, followed by re-plating of both single cells and residual small aggregates in complete fresh medium.

### Flow Cytometry

For flow cytometry, tumor spheres or monolayer cultures were mechanically dissociated to single cells, washed and incubated with the appropriate dilution of control or specific antibody. Antibodies used were PE conjugated anti-CD133/1 from Miltenyi Biotec (Bergisch Gladbach, Germany), FITC conjugated anti-CD44 (eBioscience, San Diego, CA), and anti-OCT3/4 (Santa Cruz Biotechology, CA). For PE conjugated anti-CD133/1, cells were first blocked with blocking reagent for 5 min, then a 20 min incubation with antibody. For OCT3/4 expression, cells were fixed and permeabilized with FIX & PERM® reagents (Invitrogen) following manufacture's instruction, and incubated with mouse monoclonal antibody against Oct3/4 (Santa Cruz Biotechnology) for 20 min in dark, after washed with PBS, cells were then incubated in the dark with a FITC conjugated secondary antibody (Invitrogen). As negative control, cells were stained with the appropriate isotope control. For FITC conjugated anti-CD44, cells were incubated with antibodies for 20 min in dark. After washed with PBS, cells were analyzed on a FACS Calibur flow cytometer (Becton Dickinson).

### Proteasome Function Assays

Chymotryptic, tryptic, and caspase proteasome activities were measured as described previously [25] with a few minor modifications. A549, H1299 and their primary lung tumor sphere cells were washed with phosphate buffered saline (PBS) and pelleted by centrifugation. Cell pellets were sonicated in homogenization buffer (25 mM Tris [pH 7.5], 100 mM NaCl, 5 mM ATP, 0.2% (v/v) Nonidet P-40 and 20% glycerol, and cell debris were removed by centrifugation at 4°C. Protein concentration in the resulting crude cellular extracts was determined by the Micro (bicinchoninic acid) protocol (Bio Rad, Rockford, IL) with BSA (Sigma) as standard. To measure 26S proteasome activity, 100 µg of protein from crude cellular extracts of each sample was diluted with buffer I (50 mM Tris [pH 7.4], 2 mM dithiothreitol, 5 mM MgCl_2_, 2 mM ATP) to a final volume of 1 mL (assayed in quadruplicate). The fluorogenic proteasome substrates Suc-LLVY-AMC (chymotryptic substrate; Biomol International, Plymouth Meeting, PA), Z-ARR-AMC (tryptic substrate; Calbiochem), and Z-LLE-AMC (caspase-like substrate; Biomol International) were dissolved in DMSO and added to a final concentration of 80 µM. Proteolytic activities were continuously monitored in 2 hr at 37°C by measuring the release of the fluorescent group, 7-amido-4-methylcoumarin (AMC), at a fluorescence plate reader (Spectramax M2, Molecular Devices, Sunnyvale, CA) with excitation and emission wavelengths of 380 and 460 nm, respectively.

### Alamar Blue Assay

Cell viability was measured by Alamar Blue[26]. A549 and H1299 cells were seeded in 96-well plates (BD Falcon) at a density of 2×10^3^ cells per well. For tumor sphere cultures, single cell was enzymatic and mechanical dissociated. 24 h after seeding, cells were exposed to different concentrations of cisplatin or doxorubicin as indicated for 48 h. Alamar Blue (BioSource, Camarillo, CA) was added directly to the culture media at 10% of the media volume during the last 10 h of the 48 h exposure period. Fluoresces with an excitation of 544 nm and emission detected at 590 nm was measured using a microplate reader.

### Generation of Stable Cell Lines Expressing ZsGreen-cODC Fusion Proteins Using Retroviral Transduction

The retroviral expression vector pQCXIN-ZsGreen-cODC was a kind gift from Dr. Frank Pajonk (Division of Molecular and Cellular Oncology, Department of Radiation Oncology, David Geffen School of Medicine at University of California, Los Angeles, Los Angeles, CA), in which the carboxyl terminus of the murine ornithine decarboxylase (cODC) degron, the sequence that directs the starting place of degradation, was fused to ZsGreen reporter [21]. pQCXIN-ZsGreenc-ODC was co-transfected with pVSV-G into GP2-293 pantropic retroviral packaging cells (Clontech)and the retrovirus supernatant was used to infect A549 and H1299 cells. 48 hr after infection, cells were selected with G418 (Invitrogen). The accumulation of ZsGreen-cODC protein was monitored by fluorescence microscope and flow cytometry (FL-1channel).

### Generation of Subcutaneous Lung Cancer Xenografts in Nude mice

For mice xenografts, both monolayer and tumor sphere cells were trypsinized and mechanically dissociated to obtain single cell suspensions before subcutaneous injection. Six-week-old female nude mice (Harlan Lab, Indianapolis, IN) were used. Tumor sphere (1×10^4^) and monolayer (1×10^4^) cells were s.c. injected into the left and right flanks of the same mouse to evaluate tumorigenic activity. Mice were monitored daily for the appearance of subcutaneous tumors. Mice were sacrificed at day 60 and tumor tissue was collected. Tumor volume was calculated by the formula 0.52×length×width^2^. Hematoxylin and eosin staining followed by IHC analysis were performed to analyze tumor histology. Sections were also stained with antibody against β-catenin by standard IHC protocol.

### Statistical Analysis

Data are presented as means ± SD. Differences were determined by two tailed Student's t test. A P value of <.05 was considered significant.

## Results

### SP and NSP cells form tumor sphere with the same frequency in human adenocarcinoma cell lines

**Figure 1:**
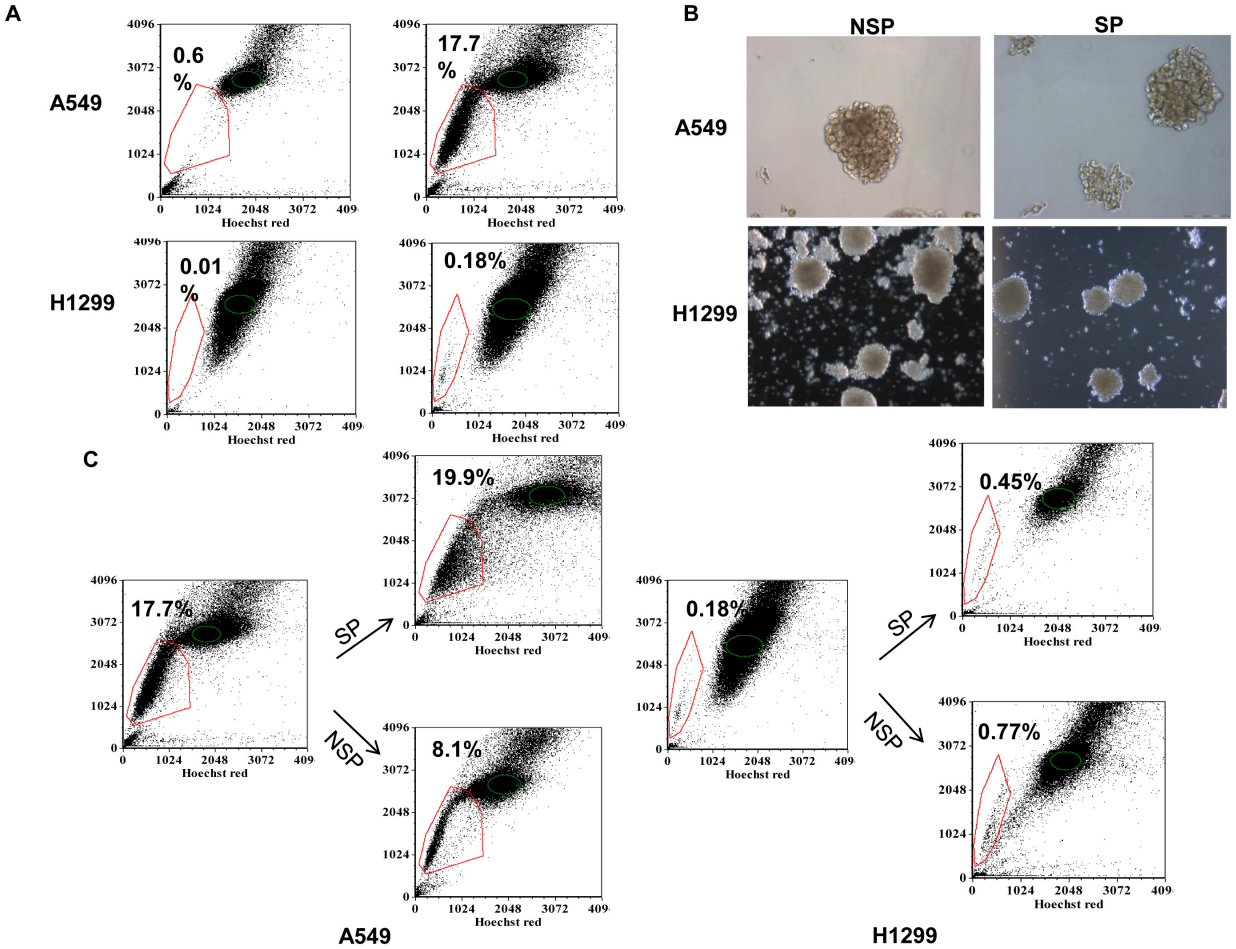
SP and NSP cells form tumor spheres with equal capacity in human adenocarcinoma cell lines. **A.** Characteristic Hoechst 33342 dye staining profile demonstrating the existence of SP (polygon gated) and NSP (ellipse gated) cells in human A549 and H1299 adenocarcinoma cells. Percentage of SP cells is indicated. Cells were stained with 5 mg/ml Hoechst33342 (HO), and control cells were also co-stained with 10 mM fumitremorgin C (FTC) to specify the SP cells. Cells were sorted using a Beckman MoFlo cytometer and data were annotated using FCS Express. **B.** Phase-contrast photographs of lung tumor spheres obtained from SP and NSP cells. SP and NSP cells (1000 cell/ml) were plated onto ultra low adherent flasks in serum free media supplemented with growth factors and cultivated as described in Material and Methods. **C.** Re-stain of SP and NSP cells from A549 and H1299 with Hoechst 33342 dye. Percentage of SP cells is labeled.

SP cells have been implicated as putative CSCs as they show elevated tumorigenicity in nude/scid mice compared to NSP cells[27]. To determine whether SP cells have greater self-renewal capacity than NSP cells in culture, SP and NSP cells from two human lung cancer cell lines were sorted based on the ability to efflux Hoechst 33342 dye to generate a Hoechst blue-red profile. A selective ABCG2 inhibitor, Fumitremorgin C (FTC), was co-incubated with Hoechst 33342 to block the activity of the transporter, and the SP gate was defined as the diminished region in the presence of FTC. The human adenocarcinoma cell lines A549 and H1299 contained 17.7% and 0.2% SP cells, respectively (Fig. 1A). Culturing of SP and NSP cells in tumor sphere forming media, which only allows for selection of undifferentiated clones or cancer stem and progenitor cells [11], resulted in lung tumor sphere formation from both subpopulations with no difference in the rate of formation (Fig. 1B and data not shown). SP cells typically give rise to SP and NSP cells by means of asymmetric cell division, whereas NSP cells do not have this potential [28]. By re-staining the sorted SP and NSP cells from A549 and H1299 cells one week after, FACS analysis revealed that the SP cells gave rise to a significant proportion of NSP cells. In addition, isolated NSP cells also gave rise to similar proportion of SP cells (Fig. 1C).

### Lung tumor spheres exhibit cancer stem cell features

**Figure 2:**
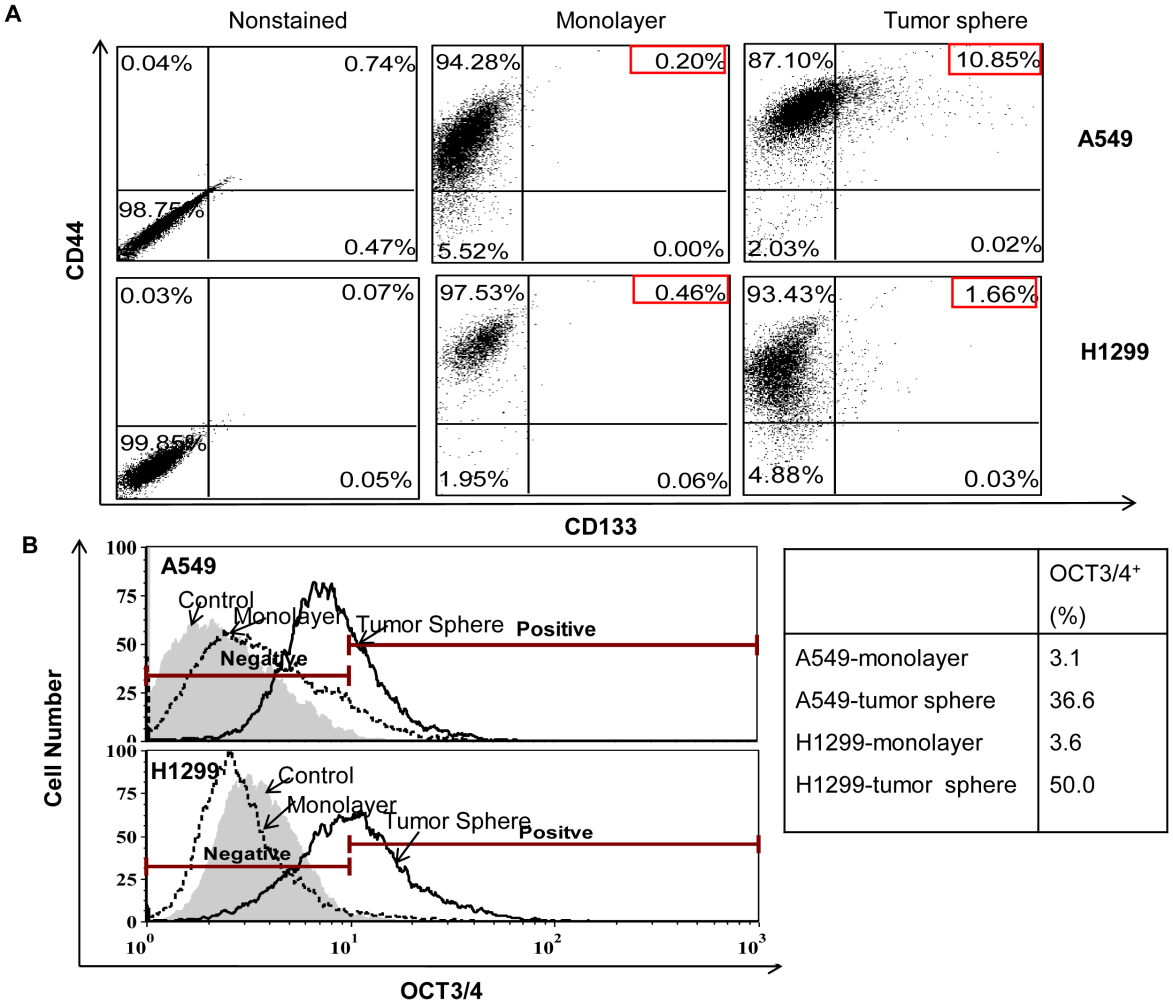
Lung tumor spheres exhibit cancer stem cell features. **A.** Lung tumor spheres showed increased CD133 expression. Expression of CD44 and CD133 in monolayer and tumor sphere cells, as determined by two-color flow cytometry analysis. Isotype-matched monoclonal antibodies and a preimmune second antiserum were used as negative controls. The percentage of single- and double-positive cells are indicated. **B.** Lung tumor spheres showed increased OCT3/4 expression. An average fluorescence intensity of OCT3/4 in control (grey area), monolayer cells (dotted line) and tumor spheres (solid line) is shown. Percentages of positive cells are indicated in the table.

Since cells capable of self-renewal reside in both side and non-side populations, we investigated whether tumor sphere cells showed any CSC features, such as the expression of stem cell markers, such as CD44, CD133 and OCT3/4. First, to rule out the effect of CSC media on the expression, monolayer cells grown in the CSC media were checked, and no difference was found between cells grow in CSC media and regular growth media (data not shown). Cells from monolayer and sphere cultures from A549 and H1299 cells were then harvested, and analyzed for CD44 and CD133. Over 90% of A549 and H1299 cells in monolayer cultures were CD44^+^, whereas only a very small portion of cells were CD133^+^ (0.2% in A549 cells, and 0.55% in H1299 cells.). However, in lung tumor spheres, CD133^+^ cells were greatly enriched to over 10% in A549, and 1.7% in H1299 (Fig. 2A). The transcription factor OCT3/4 is considered a central regulator of human embryonic stem cell pluripotency and self-renewal capacities. Its expression appears to be important in maintaining the undifferentiated state of embryonal carcinoma, as well as in other cancers [29], [30]. OCT3/4^+^ cells in A549 and H1299 monolayers were less than 4%, whereas the numbers were significantly up-regulated in tumor spheres to 36% and 50%, separately.

### Lung tumor spheres have high tumorigenic potential

**Figure 3:**
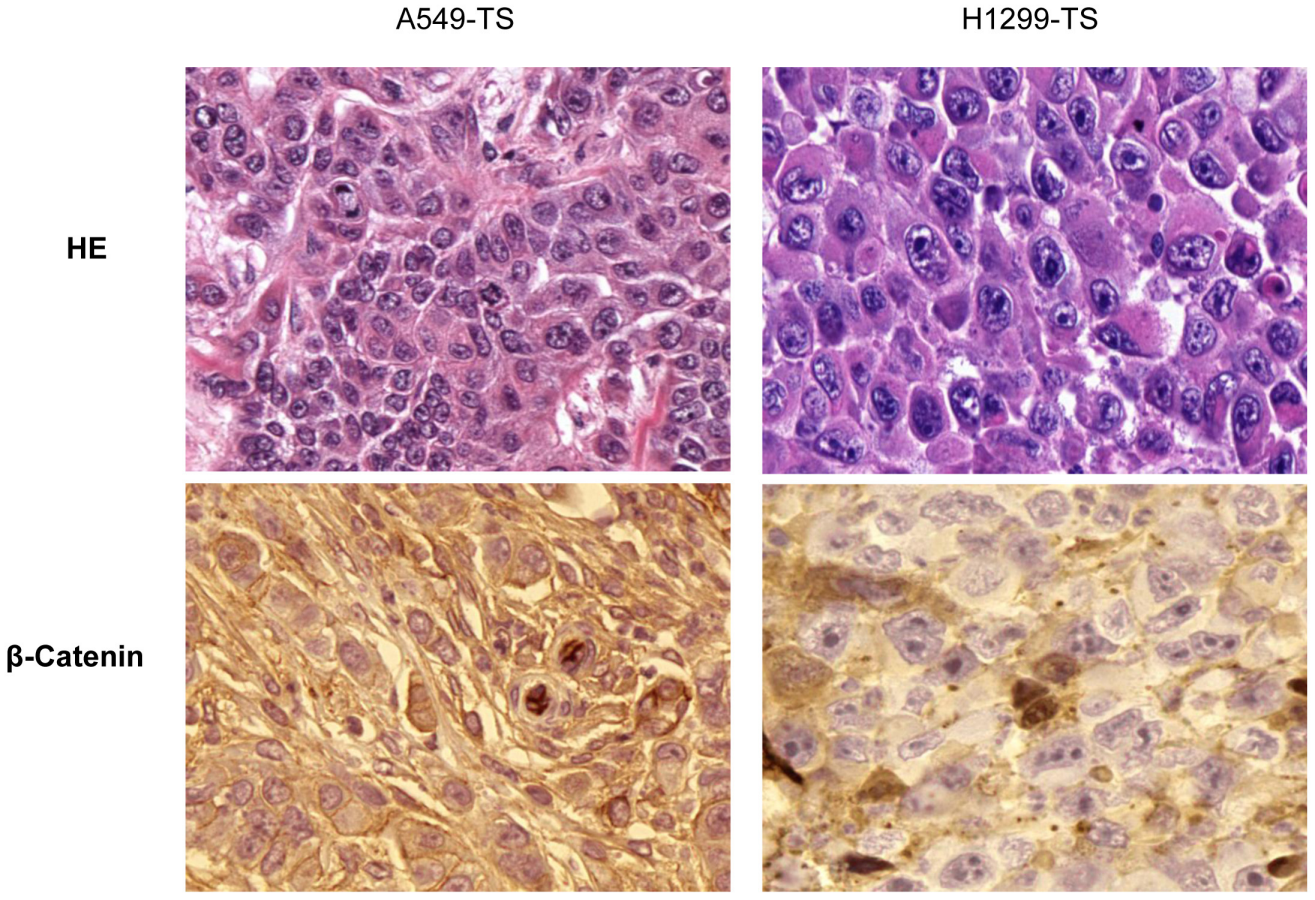
Histopathologic examination of the engrafted tumors by H&E and β-Catenin staining. Surgically removed tumor was fixed in buffered formalin and subsequently analyzed by immunohistochemistry (IHC) by standard protocol.

To determine whether lung tumor spheres from A549 and H1299 cells differ in their tumorigenic potential compared to cells grown in a monolayer, we injected 10,000 cells subcutaneously from each of these populations into the flanks of nude mice. Tumor growth was observed in all mice inoculated with tumor sphere cells derived from A549, whereas no tumor growth was observed after inoculation with monolayer A549 cells (Table 1). Tumor sphere cells from H1299 cells grew in 2 out of 3 nude mice, whereas the same number of monolayer cells failed to give rise to any tumor even with an extended 4 more weeks of observation (Table 1 and data not shown). H&E staining revealed a highly cellular mass with large nuclei and prominent nucleoli (Fig. 3). Tumor sections from xenografted tumor spheres were stained using an antibody against β-catenin, which is a key regulator of the Wnt pathway which functions in controlling stem cell self-renewal. The translocation of β-catenin to the nucleus leads to the enhanced expression of β-catenin target genes that promote tumor establishment, growth, and invasion [31]. We observed that β-catenin was expressed mainly in the cytoplasm, whereas expression in the nuclei was observed in tumor sphere induced tumor sections (Fig. 3). Translocation of β-catenin to the nucleus leads to the enhanced expression of β-catenin target genes that promote tumor establishment, growth, and invasion [31].

**Table 1:** Lung tumor spheres have high tumorigenic potential.

	A549		H1299	
	monolayer	Tumor Sphere	monolayer	Tumor Sphere
Tumor formation	0/3	3/3	0/3	2/3
Tumor size (mm^3^)		438		1011

Tumorigenic potential of tumor sphere cells is greater than monolayer cancer cells. Monolayer and tumor sphere cells from A549 were injected s.c. into athymic nude mice at concentrations of 1×10^4^ cells/ml. Mice were sacrificed at day 60, and tumor was surgically removed and measured, and the mean values of the tumor size were shown in the table.

### Lung tumor spheres are more resistant to chemotherapeutic agents

**Figure 4:**
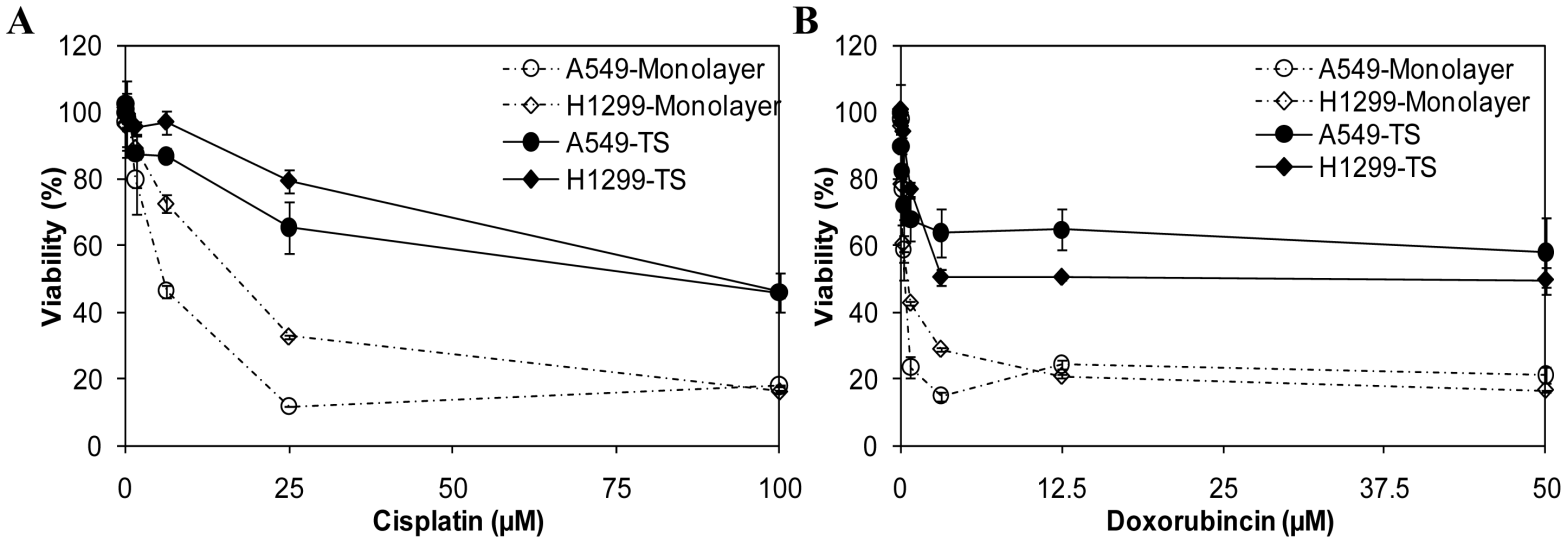
Chemoresistence of monolayer and tumor sphere. **A.** cisplatin, **B.** doxorubicin resistance of monolayer culture and tumor spheres were analyzed. Cells dissociated from lung tumor spheres or monolayers were plated out in 96-well plate at 2×10^4^ cells/ml, 24 h later, media containing different concentrations of cisplatin or doxorubicin was added. After 48 h culturing, 10% Alamar Blue was added and read at 544/590 nm, the percent viability was calculated relative to cells not exposed to any chemotherapeutic drugs. The results represent the means ± SD.

Monolayer and tumor sphere cells were exposed to different concentrations of chemotherapeutic drugs currently in use in the clinical setting, such as cisplatin and doxorubicin. Cell viability was measured by Alamar Blue assay 2 days after subjecting monolayer and tumor sphere cells to cisplatin or doxorubicin. As shown in Fig 4, the viability of monolayer cultures decreased significantly after both chemotherapeutic agents treatments compared to that of tumor sphere cells. The relative viability was around 20% for both A549 and H1299 monolayer cultures when treated with 12.5 µM of doxorubicin, while over 50% of tumor sphere cells (50.4% for H1299, and 64.8% for A549) survived. When treated with 25 µM of cisplatin, the relative viability was 11.7% for A549 and 25.3% for H1299 monolayer culture, whereas 65.4% of A549 tumor sphere and 79.3% of H1299 tumor sphere cells survived this treatment, indicating monolayer culture may be more sensitive to cisplatin and doxorubicin compared to tumor spheres, as demonstrated by the less percentage of viable cells after in vitro exposure to the drug.

### Lung tumor spheres exhibit reduced proteasome activity

**Figure 5:**
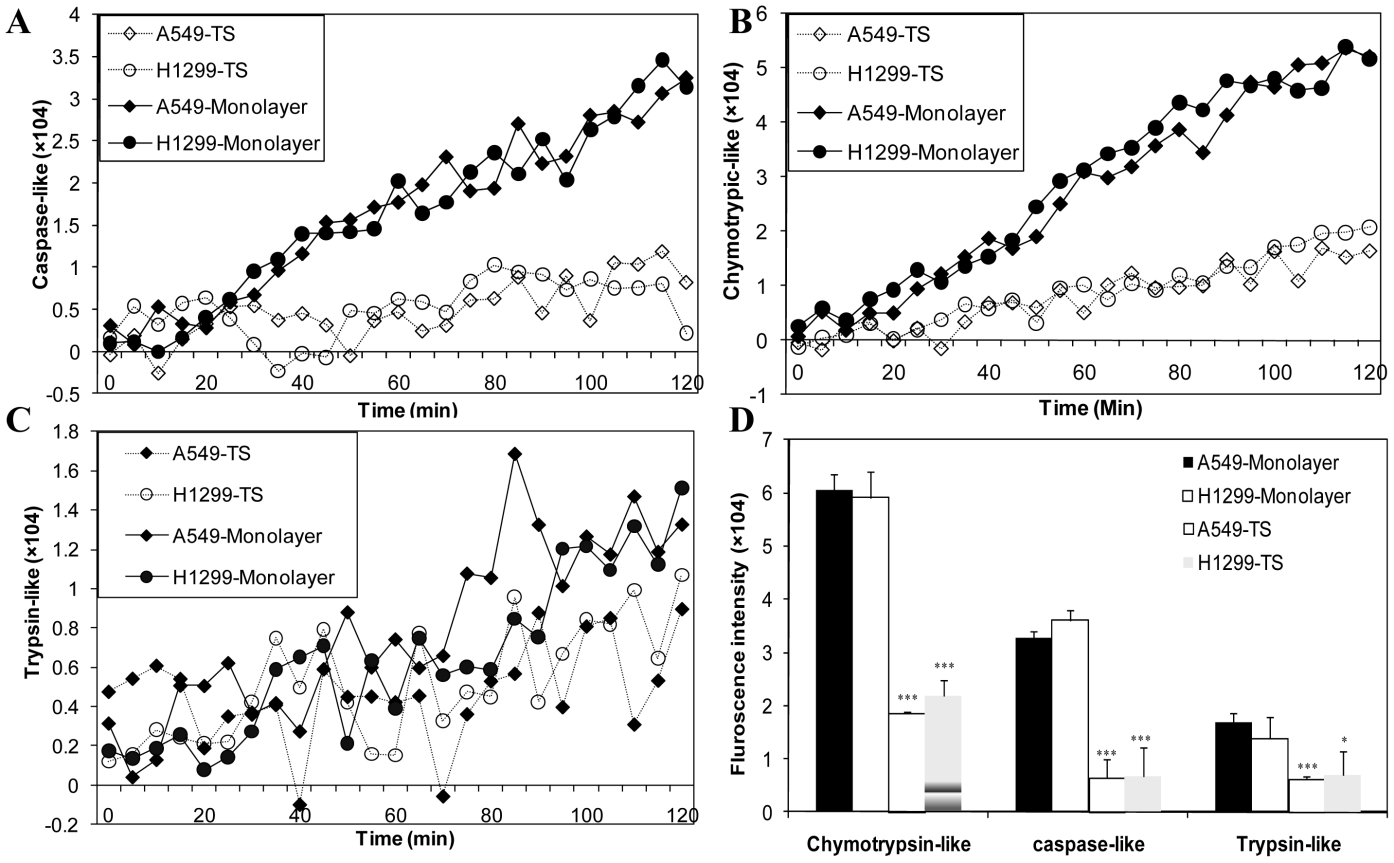
Decreased proteasome activities of the 26S proteasome in A549 and H1299 tumor spheres. **A.** caspase-like, **B.** chymotrypsin-like, and **C.** trypsin-like activity of monolayer culture and tumor spheres from A549 and H1299 cells was measued over the indicated times. **D.** End point analysis (120 min) of 26S proteasome activities in monolayer cultures and tumor spheres. Mean ± SD is plotted and derived from three independent experiments. Student's paired, and two-tailed t-tests were performed, *p<0.05, **p<0.01, and ***p<0.001 (three replicates per experiment).

Down-regulation of ubiquitin-proteasome-dependent proteolysis was observed in response to various chemotherapeutic agents, such as cisplatin [19], ifosfamide [19] and Bortezomib [32]. Recent findings showed that chemotherapeutic drugs, such as cisplatin, can also lead to propagation of CSCs [18]. To investigate whether reduced proteasome activity is observed in lung CSCs, we performed fluorogenic proteasome function assays using monolayer and sphere cultures from A549 and H1299 human NSCLC cells. The 26S proteasome has at least three distinct kinds of proteolytic activities including chymotrypsin-, trypsin-, and caspase-like activities. To monitor specific protease activities of the 26S proteasome, three fluorogenic substrates: Suc-LLVY-AMC for chymotrypsin-like, Z-ARR-AMC for trypsin-like, and Z-LLE-AMC for caspase-like activity, were incubated with cell lysates from either monolayer or lung tumor sphere cultures. Caspase-like and chymotrypsin-like activities from monolayers increased over 2 hours of incubation with substrates, but remained unchanged in tumor sphere lysates. Chymotrypsin-like activities were similarly increased in monolayer cultures and less so in lung tumor spheres (Fig. 5A, B). Trypsin-like activity was not significantly different in both groups (Fig. 5C). Chymotrypsin-like activity, the predominant activity of the 26S proteasome, was reduced to about 30% in the sphere cultures relative to monolayer cultures. Caspase-like activity was reduced to about 20%, whereas the trypsin-like activity was not significantly different (Fig. 5D). This result indicates that lung tumor sphere cultures have lower 26S proteasome activity than monolayer cultures.

### Lung tumor spheres are enriched with cells with low proteasome activity

**Figure 6:**
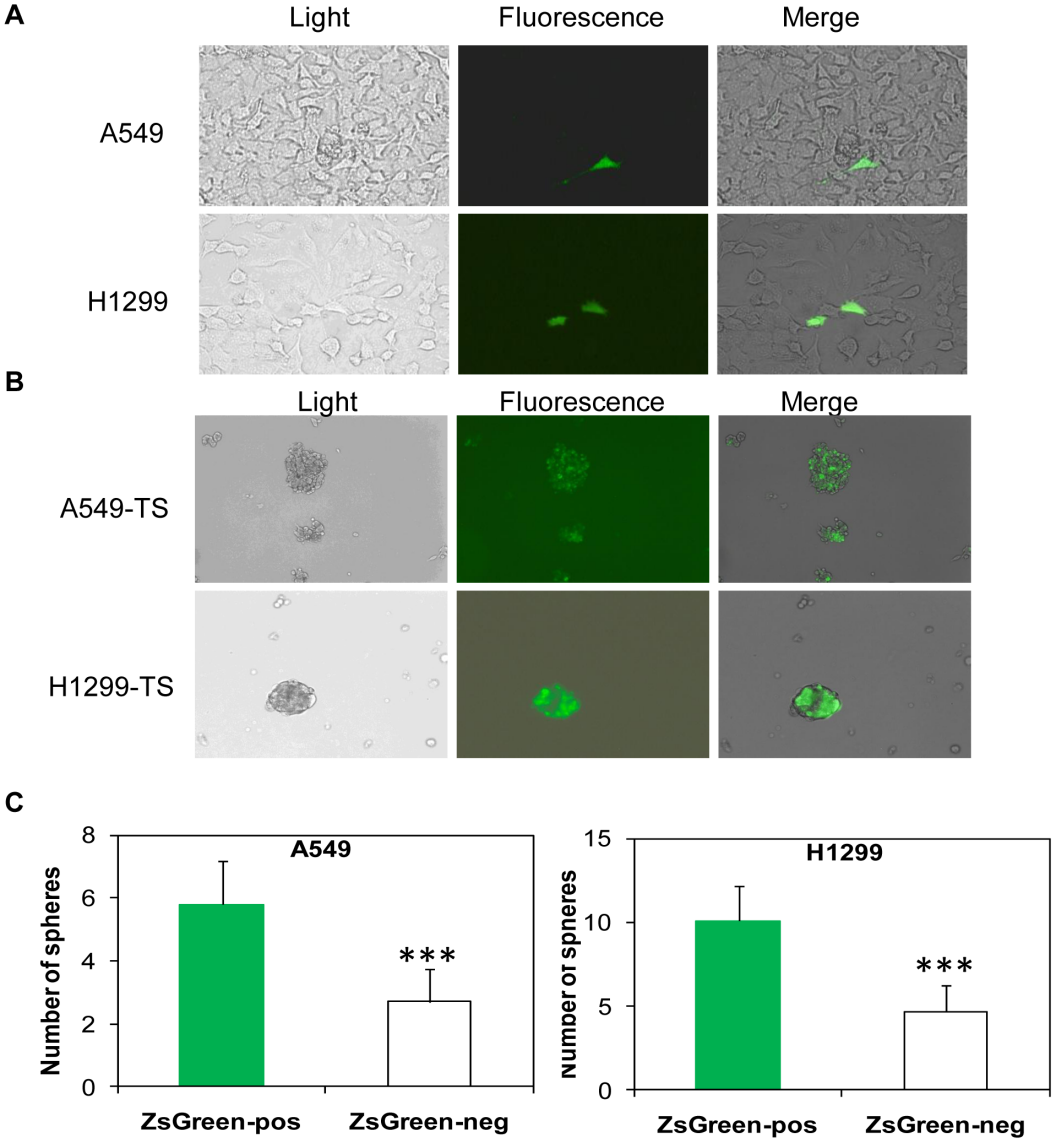
Track CSCs in tumor sphere based on their proteasome activity. **A.** Frequency of ZsGreen cells in A549 and H1299 monolayer cultures. **B.** Frequency of ZsGreen cells in A549 and H12199-derived tumor spheres with accumulation of ZsGreen-cODC, and thus low proteasome activity is indicated. **C.** Number of spheres formed from the ZsGreen positive and the ZsGreen negative cells after sorting with flow cytometry into 96-well plates. ZsGree-positive and –negative cells were sorted into 96-well plate (100cells/well), after cultured in tumor sphere media for 2 weeks; number of formed spheres was counted. Student's paired, and two-tailed t-tests were performed, ***p<0.001 (three replicates per experiment).

Proteasomal degradation of most proteins must first go through the ubiquitination pathway before being degraded by the proteasome. However, ornithine decarboxylase (ODC) is a well-characterized cellular protein subject to ubiquitin-independent proteasomal degradation, and once recognized by 26S proteasome, it leads to immediate degradation of the proteins that contain it [21]. Retrovirus expressing ZsGreen-cODC (i.e. fusion of a fluorescent protein ZsGreen, and the C-terminus of the murine ornithine decarboxylase (cODC) degron) permits identification of cells with reduced 26S proteasome activity due to ZsGreen fluorescence. This has previously been reported to track CICs in glioma and breast cancer *in vitro* based on its capability to label live cells with low 26S proteasome activity [21]. To confirm that tumor sphere cells are enriched for lung CSCs, we infected A549 and H1299 cells with the ZsGreen-cODC expressing retrovirus. We observed that monolayer cultures of A549 and H1299 exhibited low background fluorescence in more than 98% of the cells, and only very few cells (2%) displayed high levels of ZsGreen (Fig. 6A and data not shown). Interestingly, lung tumor spheres derived from ZsGreen-cODC infected A549 and H1299 cells were greatly enriched for ZsGreen-positive cells, indicating low proteasome activity and thus a highly enriched source for CSCs (Fig. 6B). To further confirm that CSCs are enriched in the population with low proteasome activity, ZsGreen-positive and –negative cells were also sorted into 96-well plate, and the number of spheres formed out of 100 sorted cells were counted, ZsGreen-positive cells from both A549 and H1299 cell lines had a statistically significantly higher sphere-forming capacity compared to the ZsGreen-negative cells (Fig.6C).

## Discussion

In the present study, we have shown that: 1) consistent with previous evidence [11], CSC-like cells do exist in lung cancer; 2) both SP and NSP cells form tumor spheres with equal frequency indicating the existence of self-renewal CSC-like cells in both populations; 3) lung CSC-like cells can be propagated in serum-free media; and 4) low 26S proteasome activity is associated with lung CSC-like cells. The cancer stem cell hypothesis predicts that the SP fraction should be able to regenerate both the SP and NSP fractions whereas the NSP fraction should only be able to regenerate itself [28]. Interestingly, the results presented here demonstrate that both the SP and the NSP fractions have the capacity to completely regenerate both fractions. The same finding has also been observed in C6 glioma and DAOY medulloblastoma cell lines where both fractions were capable of reconstituting the parental cellular population [16], [33]. In the present study, SP cells displayed tumor sphere-like growth, the classical *in vitro* assay of self-renewal potential, thereby confirming previous findings that SP cells display stem-like activity [34]. However, NSP cells were also able to form tumor spheres at a frequency comparable to that of SP cells.

CD133 (human prominin-1), a five transmembrane domain glycoprotein, was originally identified as a cell surface antigen present on CD34^+^ hematopoietic stem cells. Recently, CD133 was shown as a putative surface marker of cancer stem cells in many solid tumors, such as brain tumor [35], prostate cancer [36], colon cancer [8], ovarian cancer [37] and lung cancer [11]. The extremely low abundance of CD133^+^ or Oct3/4 ^+^ cells in tumors, makes it difficult to isolate these populations. In our study we show that enrichment of CD133^+^/Oct 3/4^+^ cells by *in vitro* serum-free culture is possible.

It has long been known that not every tumor cell is a tumor-initiating cell, and thus millions of tumor cells are often required to transplant a new tumor from an existing one. In the present study, xenograft tumor growth in nude mice occurred with injection of 10,000 tumor sphere cells, but not by injecting an equal number of monolayer cells. These tumors had large nuclei with prominent nucleoli and nuclear staining of β-catenin, indicating possible activation of Wnt pathway. Nuclear accumulation of β-catenin [38] has been shown to play a role in controlling stem cell self-renewal [31], [39].

Chemotherapeutic drugs, such as cisplatin and ifosfamide [19], are known to kill the bulk of tumors by down-regulating ubiquitin-proteasome-dependent proteolysis [20]. However, they also lead to the propagation of CSCs [18]. Decreased 26S proteasome activity was reported as a feature of CSCs in glioma and breast cancer cells, and this feature may be used to identify, track, and target CSCs *in vitro* and *in vivo* [21]. Here we present novel observations that chymotrypsin-like and caspase-like activities of the 26S proteasome were significantly lower in lung cancer cell tumor spheres compared to monolayer cultures. This suggests a new target for drug intervention.
